# Advances in the Use of Beneficial Microorganisms to Improve Nutritional and Functional Properties of Fermented Foods

**DOI:** 10.3390/foods13010155

**Published:** 2024-01-02

**Authors:** Palmira De Bellis, Carlo Giuseppe Rizzello

**Affiliations:** 1 Institute of Sciences of Food Production (ISPA), National Research Council (CNR), Via G. Amendola 122/O, 70126 Bari, Italy; 2 Department of Environmental Biology, Sapienza University of Rome, Piazzale Aldo Moro 5, 00185 Rome, Italy; carlogiuseppe.rizzello@uniroma1.it

The World Health Organization [1] has highlighted the need to improve the nutritional and functional characteristics of foods and beverages in order to enhance quality of life and prevent chronic diseases. Many foods often present critical issues such as high glycemic response, low biological protein value, high salt and fat concentrations, a lack of functional compounds such as fibers and polyphenols, and the presence of ingredients associated with hypersensitivity reactions. The use of beneficial microorganisms such as lactic acid bacteria (LAB) is an excellent strategy to improve the nutritional and functional properties of foods via the synthesis of bioactive compounds or through the degradation of antinutritional factors. In recent years, many microorganisms with metabolic characteristics useful for improving traditional and novel fermented foods have been identified, and the relationships between their application and fermented food quality, safety, and health promoting features have been elucidated.

In this Special Issue, an overview is provided of the latest scientific evidence on improving the nutritional and functional properties of food resulting from the use of beneficial microorganisms. Recent increases in scientific efforts have resulted in the developed of both new and traditional fermented products that combine the beneficial characteristics of microbial fermentation with the nutritional properties of animal- and plant-derived matrices.

In detail, bioprocessing techniques (sprouting and sourdough fermentation) applied to nonwheat grains, such as barley and lentil, can produce ingredients able to improve the technological and nutritional properties of fortified wheat breads. The modulation of fermentation parameters (native or sprouted cereal and legume flour, DY, and temperature) can lead to the production of a dextran-enriched sourdough that is suitable for the production of bread with enhanced nutritional quality (low HI and pGI), functionality (high soluble and total fiber content), and sensory attributes [2]. Some aspects related to the production of EPS by a *Weissella cibaria* strain were explored in depth by De Bellis et al. [3]. The strain selected as a high-EPS producer in the presence of sucrose was used to produce an EPS-enriched sourdough suitable for use as a fat replacer in baked goods [4]. The authors characterized the EPS produced by *W. cibaria* C43-11 and investigated the possible genetic regulatory elements responsible for the modulation of *dextransucrase (dsr)* gene expression [3]. 

Sourdough and bread with improved features were developed using fermented water extracts from Asian pears and Assam tea leaves with co-cultures of *Lactiplantibacillus plantarum* and *Saccharomyces cerevisiae* strains as starter cultures. In particular, a sourdough bread supplemented with fermented water had 10% less sugar, 12% higher dietary fiber, and two to three times higher total phenolic content and antioxidant activity than common sourdough bread [5].

Included in this Special Issue, a study addressed the microbial diversity associated with different flatbread doughs leavened with traditional brewer’s yeast or type II sourdough. The latter was started with a strain of *Leuconostoc citreum*, which was selected for the production of “yeast-free” bread. The authors explored, using an in-depth metagenetic approach, the bacterial and fungal microbiota of the dough, highlighting how the use of a microbial starter profoundly influenced the composition of the microbiota of the dough, which was directly responsible for the product quality [6].

The promising results reported through several studies indicate a possible market exploitation of innovative fermented beverages. In particular, He et al. [7] found that oats are a suitable fermentation substrate. They evaluated the effects of fermentation on the content of the bioactive components of oats, such as β-glucan, polyphenols, flavonoids, and volatile compounds. The results differed depending on the strains used in oat fermentation, indicating that oats can be used for the production of fermented beverages, providing alternative products for vegetarians and those who are lactose-intolerant.

Demarinis et al. [8] identified a suitable combination of starters and legume grains that can be used to produce a legume-based milk substitute containing high concentrations of free peptides and amino acids. These lupin- and pea-based beverages may also be carriers of probiotic LAB.

Soy drinks are an alternative to milk, especially for those who are allergic to milk proteins, lactose-intolerant, or follow a vegan diet. However, the bean flavor of soy beverages is the main factor limiting consumer acceptance. Sun et al. [9] proposed the use of a Chinese indigenous edible and medicinal fungus, *Naematelia aurantialba*, to produce a fermented soybean beverage with improved health-benefitting characteristics and a weaker beany taste compared with typical soy beverages, which are essential for its commercialization [9].

Researches have focused on the production of new fermented foods with functional properties, the consumer demand for which is increasing. Canonico et al. [10] produced a craft beer with functional properties through the conjunction of a legume-fortified beer with functional yeasts. In particular, this craft beer was fortified with hydrolyzed red lentils using selected nonconventional yeasts (*Lachancea thermotolerans* and *Kazachstania unispora*) through both pure and mixed fermentation. 

The management of food waste and by-products is crucial for the agri-food industry both for managing the costs relating to disposal and to meet current environmental regulations. Cheese whey represents the main by-product of the dairy industry; however, this whey is also a source of functional and bioactive compounds, in particular, proteins and peptides. A biotechnological protocol aimed at the valorization of exhausted ricotta whey enriched the substrate in bioactive peptides, thus creating a supplement to produce a functional ricotta [11]. 

Finally, Ha et al. [12] performed the fermentation of *Tenebrio molitor* larvae by *Cordyceps militaris* mycelium to increase the contents of nutrients such as total protein, total fiber, β-glucan, and cordycepin contents. The proposed method could allow the bio-fortification of several foods, which is crucial for populations that do not have a proper and balanced diet.

This Special Issue also includes an in-depth review on the potential of LAB as bio-preservatives through the production of various antifungal metabolites [13]. Foods are highly susceptible to spoilage due to numerous fungi, which cause economic and production losses and represent a toxicological risk to humans through the production of mycotoxins. Furthermore, consumers are increasingly demanding healthier foods that are prepared with the minimal use of chemical preservatives. Therefore, the use of LAB as biopreservatives is among the safest strategies to inhibit the growth of fungi in foods, resulting in a lengthening of their shelf life.

In summary, this Special Issue provides insights into the recent application of beneficial microorganisms to improve nutritional and functional properties of various fermented foods. We hope that readers will find this Special Issue interesting.
